# Deleterious and Oncogenic Mutations in the *IL7RA*

**DOI:** 10.3390/cancers11121952

**Published:** 2019-12-05

**Authors:** Lívia Weijenborg Campos, Leonardo Granato Pissinato, José Andrés Yunes

**Affiliations:** 1 Centro Infantil Boldrini, Campinas, SP 13083-210, Brazil; livia.w.campos@gmail.com (L.W.C.); leonardo.pissinato@gmail.com (L.G.P.); 2 Graduate Program in Genetics and Molecular Biology, State University of Campinas, Campinas, SP 13083-210, Brazil; 3 Medical Genetics Department, Faculty of Medical Sciences, State University of Campinas, Campinas, SP 13083-894, Brazil

**Keywords:** IL7RA, leukemia, SCID, Multiple Sclerosis, polymorphisms, oncogenic mutations

## Abstract

Interleukin 7 (IL-7) is a critical cytokine that plays a fundamental role in B- and T-cell development and in acute lymphoblastic leukemia (ALL). Its receptor (IL7R) is a transmembrane heterodimer formed by the IL7Rα and the IL2Rγ chain (γc). The IL7R signals through the JAK/STAT pathway. Loss-of-function mutations and some polymorphisms of the IL7Rα were associated to immunodeficiency and inflammatory diseases, respectively. Gain-of-function mutations were described in T-cell ALL and in high risk precursor B-cell ALL. Most confirmed loss-of-function mutations occur in the extracellular part of the IL7Rα while oncogenic mutations are exclusively found in the extracellular juxtamembrane (EJM) or transmembrane regions. Oncogenic mutations promote either IL7Rα/IL7Rα homodimerization and constitutive signaling, or increased affinity to γc or IL-7. This work presents a review on IL7Rα polymorphisms/mutations and attempts to present a classification based on their structural consequences and resulting biological activity.

## 1. Introduction

**Figure 1:**
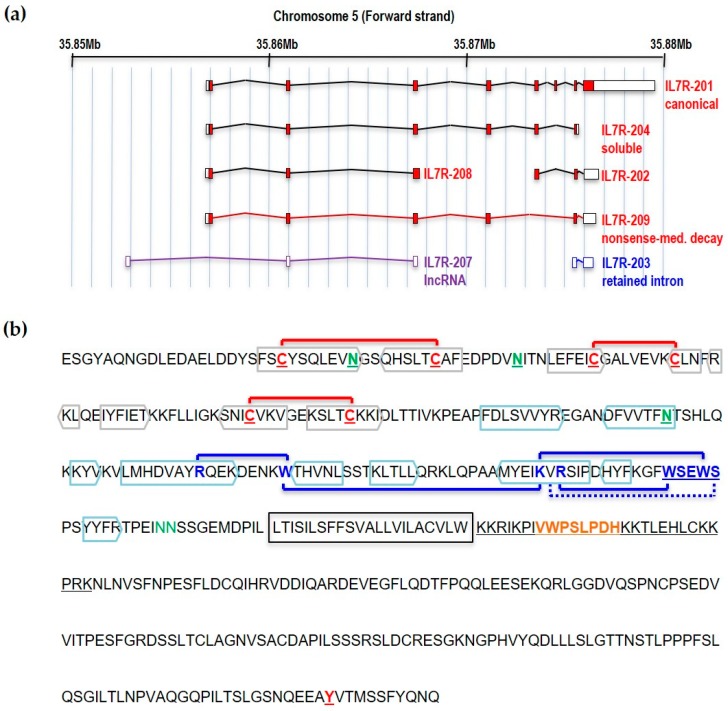
Structure of the IL7Rα gene and protein. (**a**) IL7Rα gene and different transcript isoforms. Nomenclature as in ENSEMBL, accession ENSG00000168685. Only transcripts supported by at least one Expressed Sequence Tag are shown. Filled boxes correspond to protein coding sequences. (**b**) IL7Rα amino acid sequence (without signal peptide). The extracellular part of IL7Rα consists of two fibronectin type-3 (FN3) domains: D1 and D2. Extracellular cysteines are highlighted, and SS-bonds are shown (red connecting lines). Beta sheets are boxed in grey (D1 region) or blue (D2 region) colors. The WSXWS domain, conserved in type I cytosine receptors, is shown in conjunction with cation-pi interactions (blue connecting lines) and H-bond (blue connecting dotted line). Experimentally documented (asparagine residues in green, underlined) or predicted (asparagine residues in green) N-glycosylation sites are also shown, as well as the transmembrane region (framed). JAK1 binds to the BOX1 (orange), which is part of the four-point-one protein, ezrin, radixin, moesin (FERM) domain (underlined), in the intracellular juxtamembrane region of the receptor. The tyrosine residue (Y449) proven to be important for STAT and PI3K anchoring is underlined.

IL7Rα is encoded by a gene made of eight exons located in the short arm of human chromosome five (5p13.2; coordinates 35,852,797–35,879,705 in GRCh37). There is some level of evidence (at least one mRNA or Expressed Sequence Tag) that this region of the genome is putatively transcribed into seven partially overlapping transcripts, five of which are protein coding, one encodes a lncRNA, and one is a transcript with retained intron (Figure 1a). The *IL7RA* gene is expressed at specific stages of B- and T-cell development. Although there has been some evidence of *IL7RA* expression in solid tumors from tissues other than lymphocytes, like glioma [1], breast cancer [2,3], and lung cancer [4,5,6], this evidence was not validated with orthogonal methods and in many cases were obtained from tumor-derived cell lines [7]. Information regarding the transcriptional control of the IL7Rα and its roles in B- and T-cell development may be found elsewhere [8,9,10]. In this manuscript we wish to present a review on IL7Rα mutations and polymorphisms reported so far, their structural consequences, and possible mechanisms of action.

The IL7Rα is a 459 amino acid (aa) long transmembrane glycoprotein receptor with 219 aa of extracellular domain, a single predicted 25 aa transmembrane domain and a 195 aa intracellular domain [11] (Figure 1b). Skipping of exon six by alternative splicing results in a frameshift and premature stop codon that generates a 261 aa soluble form of the IL7Rα that has been shown to potentiate IL-7 activity [12] and has been linked to autoimmune and inflammatory diseases (see below).

**Figure 2:**
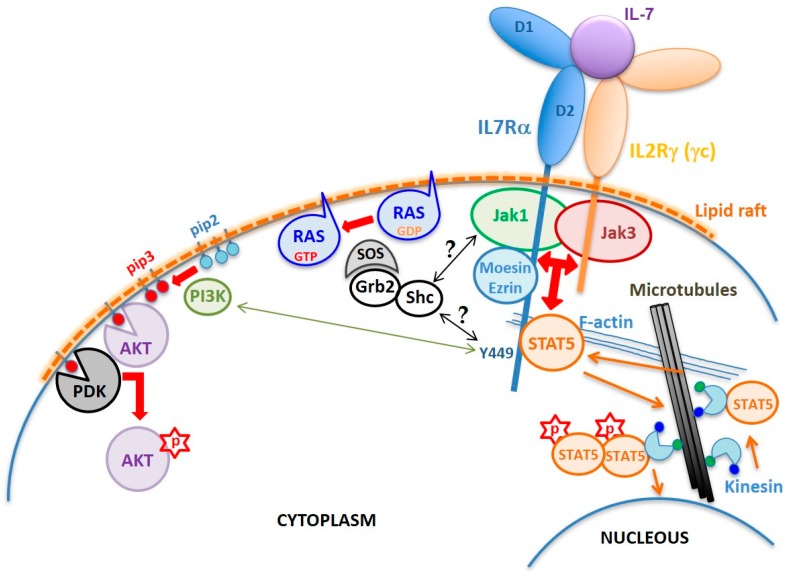
Schematic of the IL7R structure and signaling mechanism. The normal IL7R is a heterodimer formed by the IL7Rα and the IL2Rγ chain (γc) upon IL-7 binding. Interaction with IL-7 is mediated by residues corresponding to the elbow region that connects the D1 and D2 extracellular domains of the receptors. Dimerization induces the formation of cholesterol-enriched membrane microdomains (lipid rafts) and the approximation and reciprocal activation of JAK1 and JAK3, followed by phosphorylation of IL7Rα’s residue Y449. FERM proteins mediate anchoring of the receptor to F-actin. Microtubules are anchored to actin allowing translocation of STAT5 up to the membrane. Once phosphorylated, STAT5 forms dimers and is transported by kinesin along microtubules towards the nucleus where it activates transcription of different genes involved in cell survival and proliferation programs. Y449 phosphorylation also recruits and activates the PI3K pathway, which converts phosphatidylinositol (4,5)-bisphosphate (PIP2) into phosphatidylinositol (3–5)-trisphosphate (PIP3). PDK1 and AKT bind to PIP3, enabling AKT to be phosphorylated by PDK1 on S308. Complete activation of AKT requires phosphorylation on S473 by mTORC2. The mechanism of RAS activation by the IL7R is unknown, but one can suppose that tyrosine phosphorylation in the IL7R, JAK1, or JAK3 provides binding sites for adaptor proteins, such as Shc and/or Grb2, that upon phosphorylation recruit SOS to the plasma membrane, which in turn activates RAS.

As in other cytokine type I and type II receptor signaling, the IL7Rα has no kinase activity. The intracellular region of IL7Rα contains an eight aa membrane-juxtaposed domain called Box1, which binds a protein tyrosine kinase from the Janus kinase family, JAK1, which is critical for the transmission of IL7Rα signal. Activation of JAK1, however, only occurs in the presence of IL-7, that drives the heterodimerization of the IL7Rα chain with the IL2Rγ (γc) chain (Figure 2). Although IL7Rα can form dimers with γc in the absence of IL-7 [13], only in the presence of this cytokine the receptor chains come at a distance close enough to allow interaction and reciprocal phosphorylation of JAK1 (coupled to IL7Rα) and JAK3 (coupled to γc) [14]. Once activated, JAKs phosphorylate the tyrosine residue Y449 on the IL7Rα intracellular tail, allowing anchoring of STAT5, or with lower affinity STAT1 or STAT3 [15]. Once anchored in IL7Rα, STAT5 is phosphorylated by JAK (possibly JAK3), dimerizes, and translocates to the nucleus, where it activates the transcription of genes important for cell survival and proliferation [15,16,17,18]. In addition to STATs, Y449 phosphorylation also recruits phosphoinositide 3-kinase (PI3K) thus initiating another intracellular signaling cascade that leads to AKT activation [19]. Thus, IL7R triggers two major signaling cascades: JAK/STAT and PI3K/AKT [20] (Figure 2), although with different intensities depending on the cell type and its developmental stage [17,21]. In some circumstances, IL7R signaling also results in extracellular signal–regulated kinase (ERK) activation [22]. However, the exact mechanisms leading to mitogen-activated protein kinase (MAPK) and ERK activation is not known. Recent reviews have gathered some hypotheses on the mechanism of crosstalk between JAK/STAT and MAPK/ERK pathways [23,24].

The IL7Rα is also part of the receptor for thymic stromal lymphopoietin (TSLP) in which case it heterodimerizes with the cytokine receptor like factor 2 (CRLF2) chain [25]. Dimerization of IL7Rα and CRLF2, activates JAK1 and JAK2 (coupled to CRLF2) and then STATs. Curiously, the murine CRLF2 does not activate any of the four known JAKs [26] but Tec kinase [27].

## 2. Structural Determinants of the IL7Rα Activity

The extracellular part of IL7Rα consists of two fibronectin type-3 (FN3) domains: D1 and D2 (Figure 2). The elbow angle between the D1 and D2 domains is about 75°. Intramolecular disulfide bonds are only present in the D1 region: C42–C57 (common to all class I cytosine receptors), C74–C82 and C108–C118 (Figure 1b). Of the six potential N-glycosylation sites, 3 were visually observed (N49, N65, and N151) while the other three were not (N182, N232, and N233). Glycans were shown to extend away from the IL7Rα and to have no direct interaction with IL-7. Even so, IL-7 was shown to have an increased affinity to the glycosylated IL7R. It is speculated that glycosylation may affect the frequency and duration with which free IL7Rα assumes a conformation poised to bind IL-7 [28].

Crystal structure analyses revealed that IL-7 bound to IL7Rα at the receptor’s elbow region connecting the D1 and D2 domains [28]. Although there are no structures of the IL7Rα–γc heterodimer or the IL7Rα–IL-7–γc ternary complex, a structural mechanism of the IL-7 signaling was proposed by McElroy and coworkers (2012) [14]. In this model, the IL7Rα and γc are proposed to interact even prior to IL-7 binding, and this is through the elbow loop residues that connect their respective D1 and D2 domains. The extracellular part of the preformed heterodimer of IL7Rα and γc proteins would assume an “X” geometry laying onto the cell surface. Their N-and C-termini would be located on opposite ends of the “X”, in such a way that JAK1 and JAK3 attached to the intracellular domains would be kept separated by a large distance (110 Å), preventing activation. Since the IL7Rα elbow loop residues that bind to γc in the preformed heterodimer would be the same that bind to IL-7, it has been suggested that IL7Rα and γc must first dissociate to be able to bind IL-7. Upon IL-7 binding, the receptors rotate 90° away from the cell surface and this rotation brings the C-termini of IL7Rα and γc within less than 30 Å from each other so that JAK1 and JAK3 would be brought into close proximity to each other, activating signal transduction [14]. This model was based on the crystal structure of the IL-7/IL7Rα complex [28] and awaits experimental validation. Fluorescence resonance energy transfer (FRET) data published for the wild type IL2R supported the idea that approximation between IL7Rα and γc’s transmembrane α-helices is required for signaling, since they share γc [13,29,30]. However, given that JAK molecules are almost six times bigger than the intracellular portion of the IL7Rα, one may argue whether approximation is the only component responsible for signal activation. Although there may be a distance argument in keeping JAK1 and JAK3 from phosphorylating the IL7Rα intracellular domain, it is also possible to speculate that a rotational component would play a role in the transition from the inactivated to activated state of the receptor.

Like other type I cytokine receptors, the IL7Rα has a conserved WSXWS motif close to the TM region. The tryptophan residues of the WSXWS motif participate in extensive Cation-pi interactions with Trp (W178R), Lys (K204R), and arginine (R206R and R170R) side chains [28].

Besides the D1, D2 and WSXWS domains required for the correct extracellular region folding and IL-7 binding, and the intracellular Box1 and Y449 that are fundamental for JAK1 and STAT anchoring and signaling, there is the four-point-one protein, ezrin, radixin, moesin (FERM) binding domain that plays a fundamental role in anchoring of the activated IL7R receptor to the cytoskeleton. Binding of IL-7 to preformed IL7Rα-γc receptors (including JAKs) results in the inclusion of ligand bound receptors into lipid rafts of the plasma membrane, where the FERM proteins ezrin or moesin are recruited and link the receptor to F-actin. Microtubules are then anchored to the actin microfilament and grow radially from rafted receptors to the nuclear membrane. STAT5 is then carried by kinesin toward the membrane, where it binds the IL7Rα phospho-Y449, gets phosphorylated, forms STAT5 dimers, and is carried back along microtubules toward the nucleus [30].

Finally, there is the question of how long the ‘activated state’ of the IL7R chains is maintained. Binding of IL-7 to IL7R leads to rapid internalization, followed by IL7R degradation or recycling. The IL7Rα–IL-7–γc ternary complex is internalized in clathrin-coated vesicles (endosomes). Hypertonic shock of cells with 0.5 M monodansylcadaverine or 100 μM sucrose, which block the formation of clathrin vesicles, impairs IL7R signaling, indicating that IL7R signaling depends on IL7R internalization and may occur, at least partly, in the endosomes. Once internalized, IL7R degradation occurs via the ubiquitin-proteasome (can be inhibited with lactacystin) and lysosomes [31].

## 3. Deleterious Mutations in the IL7R

**Figure 3:**
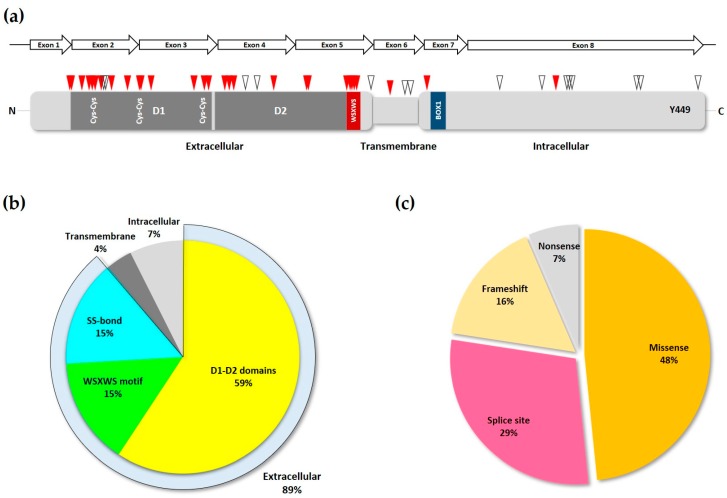
Loss-of-function IL7Rα mutations. (**a**) Schematic of IL7Rα receptor chain showing the locations of loss-of-function mutations. Red triangles represent inactivating and pathogenic mutations and polymorphisms, according to ClinVar and publications cited in the text. While empty triangles represent VUS with possible disease-causing effect as determined by computational methods. D1 and D2 domains are shown in dark grey, and the WSXWS and BOX1 domains (see Figure 1b) are represented by the red and blue boxes, respectively. Disulfide bonds in the extracellular portion of the receptor are shown by the paired Cys residues. The Y449 residue is indicated near the C-terminus portion of the protein. The *IL7RA* gene exons are represented by the arrows above the receptor protein schematic. (**b**) Frequency of inactivating mutations and polymorphisms in each of the different motifs of the IL7R chain. The extracellular portion of the receptor is represented by its three major structural domains. The SS-bond category represents the cys-cys bonds in the D1 domain. (**c**) The various types of loss-of-function mutations found in the *IL7RA* gene, and their frequency.

Deleterious, or loss-of-function, mutations in *IL7RA* have been widely described in the last few decades, most of them being associated with development of immune diseases. The IL7R receptor is crucial to T and B cell development and expansion, also it is very important for the selection of self-tolerance during T cell maturation, thus playing a major role in autoimmune diseases [32]. Most deleterious mutations in the *IL7RA* are single nucleotide variations (SNVs) resulting in amino acid changes or splice site disruption, and are concentrated in the first five exons of the gene (Figure 3a), corresponding to the extracellular domain of the transduced protein, with exon two showing the highest mutation frequency [33].

Knockout of the *IL7RA* impairs V(D)J recombination and leads to immunological deficiencies in mice [34]. Likewise, inactivating *IL7RA* mutations are strongly associated with severe combined immunodeficiency (SCID) in humans, especially the T^−^B^+^NK^+^ phenotype [35,36], with an incidence of approximately 11% of SCID cases in the US [37,38]. For example, the T allele of the *IL7RA* single nucleotide polymorphism (SNP) rs104893894 (p.P132S) was shown to be a cause of SCID. The mutant receptor has a much lower binding affinity for its ligand, IL-7, and this is a likely consequence of structural alterations in the extracellular portion of the mutant protein [36]. Other *IL7RA* mutations that result in the structural alteration of the IL7Rα and in SCID have been described [33,39,40]. A recently described IL7Rα p.F40L mutation was postulated to impair receptor function by reducing its thermo stability and expression [41].

By studying the binding interface of IL-7 with IL7Rα at the structural level, it has been demonstrated that most *IL7RA* SCID mutations locate outside of the receptor’s binding epitope, and instead are concentrated mainly in the D1, D2, and WSXWS domains of the protein. Also, some mutations affecting the cysteine participating in disulfide bonds in the D1 region have been reported. These findings suggest that, rather than affecting the receptor–ligand interaction directly, SCID mutations at these sites alter protein structural features like rigidity, stability, and folding, thereby reducing the mutant receptor affinity for the ligand in an indirect manner [28]. Analysis of the most common *IL7RA* pathogenic mutations listed in ClinVar and all other publications, showed that most of them are indeed located either in the D1, D2, WSXWS domains, or the disulfide bonds of the IL7Rα receptor (Figure 3b). Also, in agreement with Giliani and coworkers (2005) [33], the majority of mutations recorded here were missense and splice-site disrupting SNVs (Figure 3c and Table 1).

There are only two disease-associated variants found in the intracellular portion of the receptor, at position 356 (p.I356V) and 269 (p.K269fs), and they are associated with increased risk of multiple sclerosis and SCID, respectively [42,53]. Most variations with unknown significance (VUS), classified as pathogenic by computational methods [55], are commonly distributed in the intracellular portion of the receptor, although there are some in the extracellular and transmembrane portions as well. The intracellular VUS mutations, however, do not seem to overlap with Box1 or Y449 of IL7Rα (Figure 3a).

Deletions of whole exons of the *IL7RA* gene have been described in SCID cases, more specifically involving exons 2–4. These deletions inactivate the receptor function by removing entire protein domains and by generating premature stop codons that leads to the production of largely truncated proteins [42]. There are also reports of small frameshift indels at the same exons generating truncated proteins [33,46,48], and nonsense mutations, generating a premature stop codon [35,42]. Finally, some *IL7RA* SNPs and mutations are known to impair splicing of the nascent *IL7RA* transcript, thus contributing to disease also by causing frameshifts that result in protein truncations [33,42,43,45].

As mentioned before, some polymorphisms of the *IL7RA* gene have been associated with an increased risk of developing multiple sclerosis (MS), an autoimmune disease that affects the central nervous system (CNS). MS is characterized by demyelination of nerve fibers of the CNS, caused by autoreactive T cells responsive to myelin antigens [56]. The most largely studied polymorphism in the *IL7RA* gene, named SNP rs6897932 (p.T244I), is strongly associated with an increased risk of developing MS [49,50]. The T allele of this SNP, coding for an isoleucine residue at position 244, is related to the protective state, while the C allele, coding for a threonine and being the most frequent allele (approximately 77%), relates to increased susceptibility to MS with an odds ratio of around 1.2 [57]. About 56% of MS patients are homozygous for the C allele, compared to 49.2% of healthy controls [49]. It is suggested that the C allele of rs6897932 increases exon 6 skipping during *IL7RA* alternative splicing, via augmentation of an exonic splicing silencer (ESS), thus increasing the soluble form of the protein (sIL7Rα) by approximately two-fold [50]. This claim is supported by the fact that, in relation to healthy individuals, patients with MS show reduced expression of membrane-bound IL7Rα [58]. The mechanism that links sIL7Rα to MS has not been completely elucidated. It is postulated that changes in the IL7Rα/sIL7Rα ratio affect IL-7 signaling and T cell reactivity to myelin proteins [59]. The soluble isoform of the IL7Ra was shown to enhance the plasma IL-7 bioactivity, and could therefore lead to increased expansion of autoimmune cells [12]. The RNA helicase DDX39B was shown to regulate *IL7RA* mRNA splicing by enhancing the inclusion of exon six and consequently repressing the production of the sIL7Rα isoform [60].

In addition to SCID and MS, polymorphisms in the *IL7RA* gene are also shown in association with other immune diseases, such as the involvement of rs6897932 in type 1 diabetes (T1D) [51] and rheumatoid arthritis (RA) [52], rs193922641 (p.C118Y) in Omenn syndrome (OS) [39], rs3194051 (p.I356V) in T1D [51], rs1494555 (p.V138I) in graft versus host disease (GvHD) [47], rs10213865 (intronic) in sarcoidosis [54], rs11567764 (p.K187=) in tuberculosis [43], and rs1494558 (p.I66T) in IgA nephropathy (IgAN) [44] (Table 1).

## 4. Oncogenic Mutations in the IL7R

So far, all *IL7RA* mutations previously associated with oncogenic disease have been gain of function mutations. Oncogenic *IL7RA* mutations were described by our group and others [61,62,63,64,65,66] in approximately 9% of T-ALL cases [61], 7.8% of early T phenotype ALL (ETP-ALL) cases [67], 2%–3% of B-ALL cases [62,68] and 12% of Ph-like B-ALL subtype [68,69,70]. Oncogenic mutations are almost always in exon six and appear to be more common (two-fold) in children than in adults [67,68,71]. Some *IL7RA* mutations were also found in solid tumors like rectal carcinoma with high microsatellite instability (p.R267>GfsX28) and lung adenocarcinoma (p.I245>N) [65], but the contribution of these mutations to tumorigenesis is less clear. In T-ALL, the oncogenic signaling downstream IL7Rα mutations could be therapeutically explored by using JAK inhibitors [60] or a combination of PI3K-AKT and MEK inhibitors [24]. More recently, monoclonal antibodies directed against the extracellular portion of the IL7Rα were shown to inhibit T-ALL xenografts [72,73]. However, there is no proof that ALL cells having IL7Rα mutations are more sensitive to JAK/PI3K-AKT/MEK inhibition or to anti-IL7Rα antibodies than ALL or normal T-/B-cells with a wild type receptor.

Overall, the most common IL7Rα mutations are complex insertions/deletions in the extracellular juxtamembrane region. In rare cases only, indels occur more deeply into the transmembrane region of the receptor. The large size of these indel mutations may suggest participation of the terminal deoxynucleotidyl transferase (TdT) and V(D)J recombination machinery, which has been implicated in ALL mutagenesis [74,75]. We have searched for RAG cryptic recombination signal sequences (cRSS) in the IL7RA and found that, close to the mutational hot stop in exon six, there is one cRSS (5′ TTTTTCTCTGTCGCTCTGTTGGTCATCTTGGCCTGTGTG 3′) that may be a target of aberrant RAG-induced double-strand breaks (DSBs). Although recombination by RAG proteins normally require two RSSs to occur, a recently described mechanism called “cut-and-run” provided evidence that excised signal circles (ESCs), the by-product of V(D)J recombination, can bind to single cRSSs and induce DSBs at distinct genomic loci via the RAG mechanism [76].

**Figure 4:**
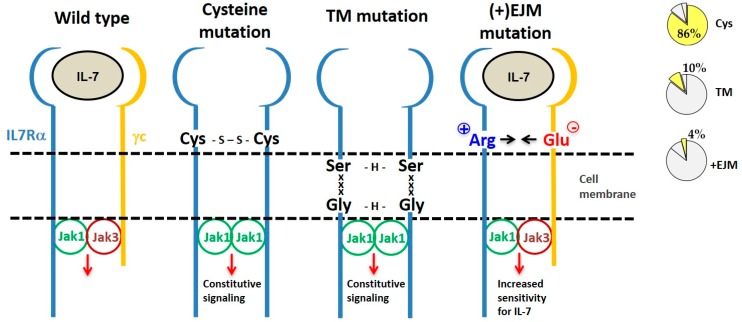
Different types of IL7Rα gain of function mutations. From left to right we show the wild type IL7R receptor and three different types of IL7Rα mutations, grouped according to their mechanism of action. Frequency of each type of IL7Rα mutations in ALL is shown in the pie charts (right). The most common IL7Rα mutations are those with cysteine insertions in the extracellular juxtamembrane (EJM) region of the receptor, which via a disulfide bond generate a stable and constitutively active IL7Rα–IL7Rα homodimer. Transmembrane (TM) mutations also result in IL7Rα–IL7Rα homodimer formation, this time by means of H-bond among an ExxxV or SxxxG motif created by the mutation. The third type of IL7Rα mutations are those with charged residue(s) insertion in the EJM of the receptor, that interact with a negatively charged residue in the γc, thus contributing to enhanced IL7Rα–γc heterodimer formation and sensitivity to IL-7.

By considering the mechanism of action, IL7Rα gain of function mutations can be divided into three categories: (a) extracellular juxtamembrane (EJM) cysteine-driven homodimers, (b) transmembrane-driven cysteine-free homodimers, and (c) EJM charged residue-enhancement of heterodimer formation (Figure 4).

### 4.1. IL7Rα Cysteine Mutants

Eighty-eight (86%) out of the 102 different oncogenic *IL7RA* mutations published so far result in unpaired cysteine insertions (Table 2). The inserted cysteine may participate in a disulfide bond connecting two mutant IL7Rα chains. This results in stable IL7Rα homodimer formation that may signal independently of IL-7, γc and JAK3. Constitutive signaling is triggered by JAK1/JAK1 trans-phosphorylation and recapitulates all of the IL7Rα downstream pathways: JAK/STAT, PI3K/AKT, and MEK/ERK. Constitutive IL7Rα/IL7Rα signaling finally contributes to cellular transformation, increased proliferation and tumor formation [22,61,62]. Among the mutants described, only a fraction was tested, however all cysteine mutants tested until now were constitutively active.

**Figure 5:**
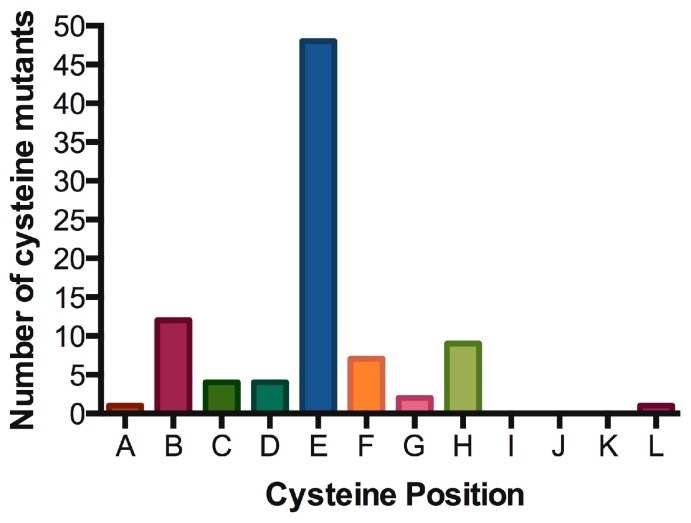
Hot spot for cysteine insertions in the oncogenic IL7Rα mutants. Preferred positions for cysteine insertions in the extracellular juxtamembrane region of the IL7Rα. For positions A to L, please refer to Table 2.

Signal transduction of cytokine receptors is dependent on (i) proximity and (ii) chain orientation, as well as (iii) time that the ‘activated state’ or dimerization of the chains is maintained [77]. The alignment of IL7Rα transmembrane segments revealed preferred positions for cysteine placement, suggestive of chain orientation restrictions. As shown in Table 2 and Figure 5, cysteine positions are concentrated in one single position (position “E” in our representation). It would be interesting to analyze whether the cysteine positioning has any impact on signaling intensity. Mutations more N-terminally would reach positions outside exon 6 and it’s “hot spot”. Mutations with cysteine inserted deeply inside the TM region (more C-terminally) are very rare, possibly reflecting the difficulty of disulfide bond formation in this chemical microenvironment. Indeed, there is a naturally occurring cysteine reside in the C-terminal end of the transmembrane domain, and the wild-type IL7Rα does not form constitutive signaling homodimers. The position of cysteine could influence the receptor chain movement up-and-down in the lipid bilayer, and eventually influence the rotation of the receptor molecules in a productive or non-productive signaling manner. In our experience, the simple presence of a cysteine and receptor homodimerization is not enough for signaling. Productive cysteine-induced intermolecular interactions probably rely on the correct alignment of the receptor’s BOX1/FERM intracellular domains, in order to allow the correct positioning and movement of JAK1/JAK1. For instance, other Type I receptors like the growth hormone receptor (GHR) or the erythropoietin receptor (EpoR) were shown to rely on correct positioning and rotation for signal activation [78,79].

The only cysteine mutation found so far outside the hot spot in exon 6 is the IL7Rα p.S185C mutation in exon five, found in six cases of ALL [62,68]. This mutation conferred proliferation advantage to Ba/F3 cells but only when co-expressed with the CRLF2 receptor, apparently by increasing the responsiveness to lower concentrations of TSLP. However, CRLF2 and IL7R-S185C did not form dimers, so the mechanism of action of this mutation cannot be attributed to disulfide bond formation and deserves further investigation [62].

### 4.2. IL7Rα Cysteine-Lacking Mutants

Cysteine-lacking mutants were described in ALL and are presented in Table 3 [61,62,64,65,80,81]. The lack of a mutant cysteine prompted the investigation of new mechanisms that would be responsible for the gain of function of these mutant receptors. Cysteine-lacking mutations can be divided into two groups according to the mutation localization: extracellular juxtamembrane (EJM) or transmembrane (TM). Cysteine lacking mutations in the TM of IL7Rα and CRLF2 receptors were studied by Shochat and collaborators (2014) [63]. Some of these mutants were proven to be capable of forming homodimers and to signal constitutively in the absence of ligand and eventually to constitutively activate JAK/STAT signaling. The activation mechanism of these cysteine-lacking TM mutants was predicted to rely on TM interactions mediated by dimerization motifs that are created by the mutant inserted residues in conjunction with some residues already present in the wild type IL7Rα. For example, in IL7Rα mutant p.253insEKV, the E residue, which is four residues N-terminally to V257 (one helix turn), forms a 253ExxxV motif that is able to form intermolecular H-bonds. Other examples of such dimerization motifs found in IL7Rα mutants are 252SxxxA and 249SxxxG. Of note, the IL7Rα p.V253G mutant chain apparently did not form homodimers, but heterodimers with the γc. However, further investigation is necessary to confirm this heterodimeric interaction. Intermolecular H-bond formation by these dimerization motifs would increase the stability of IL7Rα/IL7Rα homodimers in the absence of a cysteine or disulfide bond [63]. Depending on the spatial orientation assumed by the interacting TM helixes, the associated cytosolic JAK kinases could get closer together in a productive or nonproductive way. In fact, Shochat and collaborators (2014) [63] reported that the cysteine-lacking TM mutant IL7Rα p.V253insGEA did not activate constitutive signaling, even though they did formed homodimers.

Recently, our group focused on the second type of cysteine-lacking mutations, which are those with insertions in the extracellular juxtamembrane region of the IL7Rα. These are characterized by the presence of charged amino acids in the EJM domain, which create a positively charged electrostatic motif that facilitates interaction with a negatively charged residue in the γc [82]. Therefore, different from the previously described mechanisms, these mutants kept dependence on IL-7, γc and JAK3. It is speculated that this electrostatic interaction between the IL7Rα and γc chains produces a more stable or better-oriented IL7Rα–γc heterodimer, thus resulting in higher sensitivity to IL-7stimulation. The proliferation advantage conferred by this kind of IL7Rα mutation would be better manifested in lower IL-7 concentrations. Considering the limited amount of IL-7 available in the bone marrow microenvironment [8,83], cells expressing a mutant IL7Rα would override the population of normal cells that express the wild type IL7Rα, contributing to leukemia progression. In fact, IL-7 seems to be quite important for ALL progression [84].

## 5. Conclusions

This review presents a comprehensive description of *IL7RA* mutations found so far. The information provided by the characterization of evolutionarily conserved domains, site-directed mutagenesis, and the identification of disease-associated mutations have been of critical value to the understanding of the signaling mechanisms of this receptor. More recently, the finding of multiple and diverse activating IL7Rα mutations in acute lymphoblastic leukemia has created a unique opportunity to further study the functioning of this receptor. As shown here, there are three kinds of activating IL7Rα mutations, two of which contributing to constitutive homodimer formation and one that seems to enhance the canonical heterodimerization between IL7Rα and γc chains. We have highlighted some of the structural features that may explain receptor activation in these cases. Considering that the γc chain participates in many other cytokine receptors, which are also expressed by ALL cells, it is intriguing that oncogenic mutations are restricted to the IL7Rα. We speculate that this may result from a structure–activity relationship unique to IL7Rα or it might be simply related to the presence of a fragile hot-spot in exon six of the *IL7RA* gene. One might also consider the possibility that IL7Rα is unique in triggering a transcriptional program for lymphocyte development that gives an oncogenic advantage to the progenitor cells. These are questions that will certainly stimulate future work on this subject.

## Figures and Tables

**Table 1:** Loss-of-function mutations in IL7RA. Only pathogenic mutations and polymorphisms found in patients are displayed. The effect/possible effect, and associated disease, are specified according to the literature or ClinVar reports. The genomic positions, in chromosome 5 (GRCh38), of the intronic variants are described below their nucleotide changes.

Mutation/PolyMorphism	Nucleotide Change	Exon	Protein Site	Effect/Possible Effect	Associated Diseases
p.Q26X	c.76C>T	Exon 1	Extracellular	Premature stop-codon	SCID [42]
p.G28R	c.82G>A	Exon 2	Extracellular	Structural: Ligand affinity	SCID [33]
p.G28fsX35	c.221+2T>G	Exon 2	Extracellular	Splicing: Exon skipping	SCID [42]
p.G28fsX51	delExon2-4	Exon 2	Extracellular	Frameshift	SCID [42]
p.L35Q	c.104T>A	Exon 2	Extracellular/D1-D2	Structural: Ligand affinity	SCID [40]
p.F40L	c.120C>G	Exon 2	Extracellular/D1-D2	Protein thermo-stability	SCID [41]
p.C42Y	c.125G>A	Exon 2	Extracellular/SS-bond	Structural: Ligand affinity	SCID
p.S44R	c.132C>A	Exon 2	Extracellular/D1-D2	Structural: Ligand affinity	SCID [33]
p.V48fsX59	c.143delTG	Exon 2	Extracellular/D1-D2	Frameshift	SCID [33]
p.L55Q	c.164T>A	Exon 2	Extracellular/D1-D2	Structural: Ligand affinity	SCID [33]
p.I66T	c.197T>C	Exon 2	Extracellular/D1-D2	Splicing: Exon skipping	SCID [35]/Tuberculosis [43]/IgAN [44]
p.C74Y	c.221G>A	Exon 2	Extracellular/SS-bond	Structural: Ligand affinity	SCID [33]
p.G75fsX75	delExon3	Exon 3	Extracellular/D1-D2	Frameshift	SCID [42]
p.C82S	c.244T>A	Exon 3	Extracellular/SS-bond	Structural: Ligand affinity	SCID
p.V111=	c.333T>A	Exon 3	Extracellular/D1-D2	Splicing: Exon truncation	SCID [45]
p.C118Y	c.353G>A	Exon 3	Extracellular/SS-bond	Structural: Ligand affinity	OS [39]/SCID [45]
p.I121fsX128	c.361dupA	Exon 3	Extracellular/D1-D2	Frameshift	SCID [46]
p.P132S	c.394C>T	Exon 4	Extracellular/D1-D2	Structural: Ligand affinity	SCID [36]
p.L135R	c.404T>G	Exon 4	Extracellular/D1-D2	Structural: Ligand affinity	SCID [33]
p.V138I	c.412G>A	Exon 4	Extracellular/D1-D2	Splicing: Exon truncation	SCID [35]/GvHD [47]
p.H165=	c.495C>T	Exon 4	Extracellular/D1-D2	Splicing: Exon truncation	SCID
p.K187=	c.561G>A	Exon 5	Extracellular/D1-D2	Splicing: Exon truncation	Tuberculosis [43]
p.L188fsX188	c.562delC	Exon 5	Extracellular/D1-D2	Frameshift	SCID [48]
p.G215V	c.644G>T	Exon 5	Extracellular/D1-D2	Structural: Ligand affinity	SCID [40]
p.W217X	c.651G>A	Exon 5	Extracellular/WSXWS	Premature stop-codon	SCID [35]
p.S218N	c.653G>A	Exon 5	Extracellular/WSXWS	Structural: Ligand affinity	SCID [33]
p.W220C	c.660G>C	Exon 5	Extracellular/WSXWS	Structural: Ligand affinity	SCID [33]
p.S221I	c.662G>T	Exon 5	Extracellular/WSXWS	Structural: Ligand affinity	SCID [40]
p.T244I	c.731C>T	Exon 6	Transmembrane	Splicing: Exon skipping	MS [49,50]/T1D [51]/RA [52]
p.K269fsX269	c.876+6T>G	Exon 7	Intracellular	Splicing: Exon skipping	SCID [42]
p.I356V	c.1066A>G	Exon 8	Intracellular	Splicing: Exon truncation	MS [49,53]/T1D [51]
-	Int. A>C, T (35857748)	Intron 1	-	Unknown	Sarcoidosis [54]
-	c.83-2A>T,G (35860850)	Intron 1	-	Splicing defect	SCID [33]

**Table 2:** Oncogenic IL7Rα mutations with cysteine insertion. Residues spanning part of the extracellular juxtamembrane, the whole transmembrane and part of the intracellular juxtamembrane regions of the IL7Rα are shown. The predicted transmembrane regions (TMpred: https://embnet.vital-it.ch/software/TMPRED_form.html) are boxed. Mutants were listed according to the position of the cysteine from N to C-terminus. Insertions are shown in red. All cysteine positions are represented by letters (A to M mapped in green), Positions B, E and H are the most common ones (see Figure 5).

Protein Mutation	TM Sequence	Associated Disease
WT	YFRTPEINNSSGEMDPILLTISILSFFSVALLVILACVLWKKRIK	
	-------------**ABCDEFGHIJKLM**-------------------	
p.L242>FCTPVP	EINNSSGEMDPIF**C**TPVPLTISILSFFSVALLVILACVLWKKRIK	T-ALL [80]
p.I241_L242>insCLEG	RTPEINNSSGEMDP**CLEG**LTISILSFFSVALLVILACVLWKKRIK	T-ALL [61]
p.L242_L243insFCRKD	EINNSSGEMDPIL**FCRKD**LTISILSFFSVALLVILACVLWKKRIK	T-ALL [62]
p.L242>FDCIGV	EINNSSGEMDPI**FDCIGV**LTISILSFFSVALLVILACVLWKKRIK	T-ALL [62]
p.L242_T243>CGIREI	TPEINNSSGEMDPI**C**GIREIISILSFFSVALLVILACVLWKKRIK	T-ALL [62]
p.I241_L242>CRPH	RTPEINNSSGEMDP**CRPH**LTISILSFFSVALLVILACVLWKKRIK	T-ALL [62]
p.L242>CWMK	TPEINNSSGEMDPI**CWMK**LTISILSFFSVALLVILACVLWKKRIK	T-ALL [62]
p.P240_I241insCS	RTPEINNSSGEMDP**CS**ILLTISILSFFSVALLVILACVLWKKRIK	T-ALL [65]
p.L242>CSQI	TPEINNSSGEMDPI**CSQI**LTISILSFFSVALLVILACVLWKKRIK	T-ALL [80]
p.L243>PCAQGI	EINNSSGEMDPIL**PCAQG**ITISILSFFSVALLVILACVLWKKRIK	T-ALL [80]
p.L242delinsLCHRK	PEINNSSGEMDPI**LCHRK**LTISILSFFSVALLVILACVLWKKRIK	T-ALL [66]
p.I241>ITLYCKT	INNSSGEMDP**ITLYCKT**LLTISILSFFSVALLVILACVLWKKRIK	T-ALL [67]
p.L242>FSCGP	PEINNSSGEMDPI**FSCGP**LTISILSFFSVALLVILACVLWKKRIK	T-ALL [62]
p.L242_L243insCPS	TPEINNSSGEMDPIL**CPS**LTISILSFFSVALLVILACVLWKKRIK	T-ALL [80]
p.L243>CPSP	TPEINNSSGEMDPIL**CPSP**TISILSFFSVALLVILACVLWKKRIK	T-ALL [62]
p.T244_I245insCPDGR	EINNSSGEMDPILLT**CPDGR**ISILSFFSVALLVILACVLWKKRIK	ph-Like ALL [80]
p.L242delinsLTACQP	EINNSSGEMDPI**LTACQP**LTISILSFFSVALLVILACVLWKKRIK	T-ALL [66]
p.L243>RCPS	TPEINNSSGEMDPIL**RCPS**TISILSFFSVALLVILACVLWKKRIK	T-ALL [62]
p.T244_I245insPPVCSVT	NNSSGEMDPILLT**PPVCS**VTISILSFFSVALLVILACVLWKKRIK	B-ALL [62]
p.IL241-242TC	RTPEINNSSGEMDPI**TC**LLTISILSFFSVALLVILACVLWKKRIK	T-ALL [67]
p.L242_L243insNPC	TPEINNSSGEMDPIL**NPC**LTISILSFFSVALLVILACVLWKKRIK	T-ALL [61]
p.T244_I245insCPT	TPEINNSSGEMDPILLT**CP**TISILSFFSVALLVILACVLWKKRIK	T-ALL [61]
p.I241_T244>SANCGA	RTPEINNSSGEMDP**SANC**GAISILSFFSVALLVILACVLWKKRIK	T-ALL [61]
p.L243_T244insVSCP	PEINNSSGEMDPILL**VSCP**TISILSFFSVALLVILACVLWKKRIK	T-ALL [61]
p.P240_L242>QSPSC	RTPEINNSSGEMD**QSPSC**LIISILSFFSVALLVILACVLWKKRIK	T-ALL [61]
p.P240_T244>RFCPH	YFRTPEINNSSGEMD**RFCPH**ISILSFFSVALLVILACVLWKKRIK	T-ALL [61]
p.L242_T244>FHPFNCGP	EINNSSGEMDPI**FHPFNCG**PISILSFFSVALLVILACVLWKKRIK	T-ALL [61]
p.L243_T244insMCP	TPEINNSSGEMDPILL**MCP**TISILSFFSVALLVILACVLWKKRIK	T-ALL [61]
p.L243>RLECV	PEINNSSGEMDPIL**RLEC**VTISILSFFSVALLVILACVLWKKRIK	T-ALL [61]
p.L242_L243>WAALLNCE	INNSSGEMDPI**WAALLNCE**TISILSFFSVALLVILACVLWKKRIK	T-ALL [81]
p.L242_L243insRC	RTPEINNSSGEMDPIL**RC**LTISILSFFSVALLVILACVLWKKRIK	T-ALL [62]
p.L243_T244>PCPL	RTPEINNSSGEMDPIL**PC**PLISILSFFSVALLVILACVLWKKRIK	T-ALL [62]
p.244 Ins MPEQDCP +S246T	NNSSGEMDPILL**MPEQDC**PTI**T**ILSFFSVALLVILACVLWKKRIK	T-ALL [62]
p.E237_L242>ASWC	SYYFRTPEINNSSG**ASWC**LTISILSFFSVALLVILACVLWKKRIK	T-ALL [62]
p.L242_T244>CPP	YFRTPEINNSSGEMDPI**CP**PISILSFFSVALLVILACVLWKKRIK	T-ALL [62]
p.L243_T244>PLCSA	TPEINNSSGEMDPIL**PLCS**AISILSFFSVALLVILACVLWKKRIK	T-ALL [62]
p.L243_T244>PIYRCVL	EINNSSGEMDPIL**PIYRC**VLISILSFFSVALLVILACVLWKKRIK	T-ALL [62]
p.L242>FEC	RTPEINNSSGEMDPI**FEC**LTISILSFFSVALLVILACVLWKKRIK	T-ALL [62]
p.L242_T244>FTCPS	RTPEINNSSGEMDPI**FTCPS**ISILSFFSVALLVILACVLWKKRIK	T-ALL [62]
p.S249_F250insCSTISILS	NSSGEMDPILLTISILS**CST**ISILSFFSVALLVILACVLWKKRIK	T-ALL [62]
p.243 Ins RCI	RTPEINNSSGEMDPIL**RC**ITISILSFFSVALLVILACVLWKKRIK	T-ALL [62]
p.L242_L243insGC	RTPEINNSSGEMDPIL**GC**LTISILSFFSVALLVILACVLWKKRIK	T-ALL [62]
p.L243>GCI	RTPEINNSSGEMDPIL**GC**ITISILSFFSVALLVILACVLWKKRIK	T-ALL [62]
p.T244_I245insLPCVY	EINNSSGEMDPILLT**LPC**VYISILSFFSVALLVILACVLWKKRIK	T-ALL [62]
p.T244>KKCTN	PEINNSSGEMDPILL**KKCTN**ISILSFFSVALLVILACVLWKKRIK	T-ALL [62]
p.L243_T244insPPCL	PEINNSSGEMDPILL**PPC**LTISILSFFSVALLVILACVLWKKRIK	B-ALL [62]
p.T244_I245insCHL	TPEINNSSGEMDPILLT**CHL**ISILSFFSVALLVILACVLWKKRIK	B-ALL [62]
p.L243_T244insSRCL	PEINNSSGEMDPILL**SRC**LTISILSFFSVALLVILACVLWKKRIK	T-ALL [65]
p.M238_L243>PCK	PSYYFRTPEINNSSGE**P****C**KTISILSFFSVALLVILACVLWKKRIK	B-ALL [65]
p.L242_L243insLTARGC	INNSSGEMDPIL**LTARGC**LTISILSFFSVALLVILACVLWKKRIK	B-ALL [65]
p.T244_I245insNPPCGT	INNSSGEMDPILLT**NPPC**GTISILSFFSVALLVILACVLWKKRIK	T-ALL [64]
P.L243>RCL	RTPEINNSSGEMDPIL**RC**LTISILSFFSVALLVILACVLWKKRIK	T-ALL [80]
P.L243>RGCL	TPEINNSSGEMDPIL**RGC**LTISILSFFSVALLVILACVLWKKRIK	T-ALL [80]
p.L242_L243SRC	TPEINNSSGEMDPIL**SRC**LTISILSFFSVALLVILACVLWKKRIK	T-ALL [80]
p.T244>RRCSS	PEINNSSGEMDPILL**RRCSS**ISILSFFSVALLVILACVLWKKRIK	T-ALL [80]
p.L243>LQRCT	PEINNSSGEMDPIL**LQRCT**TISILSFFSVALLVILACVLWKKRIK	T-ALL [80]
p.T244>RGFHITCQT	NSSGEMDPILL**RGFHITCQT**ISILSFFSVALLVILACVLWKKRIK	T-ALL [80]
p.P240_T244>SCLI	YYFRTPEINNSSGEMD**SC**LIISILSFFSVALLVILACVLWKKRIK	ph-Like ALL [68]
p.L243_T244>CAN	FRTPEINNSSGEMDPIL**CAN**ISILSFFSVALLVILACVLWKKRIK	ph-Like ALL [68]
p.L243_T244>RCPP	RTPEINNSSGEMDPIL**RC**PPISILSFFSVALLVILACVLWKKRIK	ph-Like ALL [68]
p.GCinsL243	RTPEINNSSGEMDPIL**GC**LTISILSFFSVALLVILACVLWKKRIK	ETP-ALL [67]
p.L242>DTRVYNSIC	NSSGEMDPI**DTRVYNSIC**LTISILSFFSVALLVILACVLWKKRIK	ETP-ALL [67]
p.LL242-243>SPCI	RTPEINNSSGEMDPI**SPC**ITISILSFFSVALLVILACVLWKKRIK	ETP-ALL [67]
p.L242delinsLPC	RTPEINNSSGEMDPI**LPC**LTISILSFFSVALLVILACVLWKKRIK	T-ALL [66]
p.L243delinsLMCP	TPEINNSSGEMDPIL**LMCP**TISILSFFSVALLVILACVLWKKRIK	T-ALL [66]
p.L242delinsLSRPC	PEINNSSGEMDPI**LSRPC**LTISILSFFSVALLVILACVLWKKRIK	T-ALL [66]
p.P240_L242>SC	YFRTPEINNSSGEMD**SC**LTISILSFFSVALLVILACVLWKKRIK	ph-Like ALL [69]
p.L242>FPGVC	PEINNSSGEMDPI**FPGVC**LTISILSFFSVALLVILACVLWKKRIK	B-ALL [69]
p.L243_T244>RCGA	TPEINNSSGEMDPILL**RC**GAISILSFFSVALLVILACVLWKKRIK	B-ALL [69]
p.L242_L243>FPHQHC	PEINNSSGEMDPI**FPHQHC**TISILSFFSVALLVILACVLWKKRIK	T-ALL [61]
p.T244_I245insRPCG	PEINNSSGEMDPILLT**RPCG**ISILSFFSVALLVILACVLWKKRIK	T-ALL [62]
p.T244>SRCG	TPEINNSSGEMDPILL**SRCG**ISILSFFSVALLVILACVLWKKRIK	T-ALL [64]
T244>TSPPCG	EINNSSGEMDPILL**TSPPCG**ISILSFFSVALLVILACVLWKKRIK	T-ALL [80]
p.I245>TKPCII	EINNSSGEMDPILLT**TKPC**IISILSFFSVALLVILACVLWKKRIK	T-ALL [80]
p.L243_T244>RQGCP	TPEINNSSGEMDPIL**RQGCP**ISILSFFSVALLVILACVLWKKRIK	ph-Like ALL [68]
p.T244>TGPCF	PEINNSSGEMDPILL**TGPCF**ISILSFFSVALLVILACVLWKKRIK	B-ALL [69]
p.T244>NDCS	RTPEINNSSGEMDPILL**NDCS**SILSFFSVALLVILACVLWKKRIK	T-ALL [77]
p.D239_T244>SFC	YFRTPEINNSSGEM**S**F**C**ISILSFFSVALLVILACVLWKKRIK	ph-Like ALL [68]
p.P240_S246>LKC	SPSYYFRTPEINNSSGEMD**LK****C**ILSFFSVALLVILACVLWKKRIK	T-ALL [61]
p.L242_S246>PQGGC	YFRTPEINNSSGEMDPI**PQG**G**C**ILSFFSVALLVILACVLWKKRIK	T-ALL [61]
p.P240_S246>LQSC	PSYYFRTPEINNSSGEMD**LQSC**ILSFFSVALLVILACVLWKKRIK	T-ALL [61]
p.I245_S246>HRGC	RTPEINNSSGEMDPILLT**HR****GC**ILSFFSVALLVILACVLWKKRIK	T-ALL [62]
p.I245_S246>SHQPC	TPEINNSSGEMDPILLT**SHQP****C**ILSFFSVALLVILACVLWKKRIK	T-ALL [62]
p.I247>KCH	RTPEINNSSGEMDPILLTIS**KCH**LSFFSVALLVILACVLWKKRIK	T-ALL [62]
p.I241_S246>TC	SPSYYFRTPEINNSSGEMDP**TC**ILSFFSVALLVILACVLWKKRIK	ph-Like ALL [68]
p.L243_S246>RVPGC	FRTPEINNSSGEMDPIL**R****VPGC**ILSFFSVALLVILACVLWKKRIK	ph-Like ALL [68]
p.P240_S246>RAYC	YFRTPEINNSSGEMD**R****AYC**ILSFFSVALLVILACVLWKKRIK	ph-Like ALL [68]
p.L248_S251>CQ	SYYFRTPEINNSSGEMDPILLTISI**CQ**SVALLVILACVLWKKRIK	T-ALL [62]

**Table 3:** Oncogenic cysteine-lacking mutations in IL7Rα. Residues spanning part of the extracellular juxtamembrane, the whole predicted transmembrane (TMpred) and part of the intracellular juxtamembrane regions of the IL7Rα are shown. The transmembrane region is boxed. Mutations are shown in red.

Protein Mutation	TM Sequence	Associated Disease
IL7R_WT	FRTPEINNSSGEMDPILLTISILSFFSVALLVILACVLWKKRIK	-
	**TM mutations**	
p.I247_L248insQW	TPEINNSSGEMDPILLTISI**QW**LSFFSVALLVILACVLWKKRIK	T-ALL [61]
p.S252_A254>WN	YFRTPEINNSSGEMDPILLTISILSFF**WN**LLVILACVLWKKRIK	T-ALL [61]
p.V253>GPSL	PEINNSSGEMDPILLTISILSFFS**GPSL**ALLVILACVLWKKRIK	T-ALL [61]
p.V253_L254insGEA	PEINNSSGEMDPILLTISILSFFSV**GEA**ALLVILACVLWKKRIK	T-ALL [62]
p.A254_L255>EKV	RTPEINNSSGEMDPILLTISILSFFSV**EKV**LVILACVLWKKRIK	T-ALL [62]
p.V253G	FRTPEINNSSGEMDPILLTISILSFFS**G**ALLVILACVLWKKRIK	T-ALL [62]
p.F250_V253>PLGE	FRTPEINNSSGEMDPILLTISILS**PLGE**ALLVILACVLWKKRIK	T-ALL [81]
p.V253>GPLV	PEINNSSGEMDPILLTISILSFFS**GPLV**ALLVILACVLWKKRIK	T-ALL [80]
p.L256>FLEL	PEINNSSGEMDPILLTISILSFFSVALFLELVILACVLWKKRIK	T-ALL [80]
p.V253>GFSV	PEINNSSGEMDPILLTISILSFFSGFSVALLVILACVLWKKRIK	ETP-ALL [67]
	**EJM mutations**	
p.L243>RRI	TPEINNSSGEMDPIL**RR****I**TISILSFFSVALLVILACVLWKKRIK	T-ALL [65]
p.T244>RI	RTPEINNSSGEMDPILL**R****I**ISILSFFSVALLVILACVLWKKRIK	T-ALL [64]
p.L243>RRL	TPEINNSSGEMDPIL**RR****L**TISILSFFSVALLVILACVLWKKRIK	T-ALL [80]
p.I241>IH	RTPEINNSSGEMDP**IH**LLTISILSFFSVALLVILACVLWKKRIK	T-ALL [80]

## References

[1] Cui L., Fu J., Pang J.C.-S., Qiu Z.-K., Liu X.-M., Chen F.-R., Shi H.-L., Ng H.-K., Chen Z. (2012). Overexpression of IL-7 enhances cisplatin resistance in glioma. Cancer Biol. Ther..

[2] Al-Rawi M.A.A., Rmali K., Mansel R.E., Jiang W.G. (2004). Interleukin 7 induces the growth of breast cancer cells through a wortmannin-sensitive pathway. Br. J. Surg..

[3] Yang J., Zeng Z., Peng Y., Chen J., Pan L., Pan D. (2014). IL-7 splicing variant IL-7δ5 induces EMT and metastasis of human breast cancer cell lines MCF-7 and BT-20 through activation of PI3K/Akt pathway. Histochem. Cell Biol..

[4] Ming J., Jiang G., Zhang Q., Qiu X., Wang E. (2012). Interleukin-7 up-regulates cyclin D1 via activator protein-1 to promote proliferation of cell in lung cancer. Cancer Immunol. Immunother..

[5] Liu Z.-H., Wang M.-H., Ren H.-J., Qu W., Sun L.-M., Zhang Q.-F., Qiu X.-S., Wang E.-H. (2014). Interleukin 7 signaling prevents apoptosis by regulating bcl-2 and bax via the p53 pathway in human non-small cell lung cancer cells. Int. J. Clin. Exp. Pathol..

[6] Suzuki K., Kadota K., Sima C.S., Nitadori J., Rusch V.W., Travis W.D., Sadelain M., Adusumilli P.S. (2013). Clinical Impact of Immune Microenvironment in Stage I Lung Adenocarcinoma: Tumor Interleukin-12 Receptor β2 (IL-12Rβ2), IL-7R, and Stromal FoxP3/CD3 Ratio Are Independent Predictors of Recurrence. J. Clin. Oncol..

[7] Cosenza L., Gorgun G., Urbano A., Foss F. (2002). Interleukin-7 receptor expression and activation in nonhaematopoietic neoplastic cell lines. Cell. Signal..

[8] Mazzucchelli R., Durum S.K. (2007). Interleukin-7 receptor expression: Intelligent design. Nat. Rev. Immunol..

[9] Carrette F., Surh C.D. (2012). IL-7 signaling and CD127 receptor regulation in the control of T cell homeostasis. Semin. Immunol..

[10] Barata J.T., Durum S.K., Seddon B. (2019). Flip the coin: IL-7 and IL-7R in health and disease. Nat. Immunol..

[11] Goodwin R. (1990). Cloning of the human and murine interleukin-7 receptors: Demonstration of a soluble form and homology to a new receptor superfamily. Cell.

[12] Lundstrom W., Highfill S., Walsh S.T.R., Beq S., Morse E., Kockum I., Alfredsson L., Olsson T., Hillert J., Mackall C.L. (2013). Soluble IL7R potentiates IL-7 bioactivity and promotes autoimmunity. Proc. Natl. Acad. Sci. USA.

[13] Rose T., Pillet A.-H., Lavergne V., Tamarit B., Lenormand P., Rousselle J.-C., Namane A., Thèze J. (2010). Interleukin-7 Compartmentalizes Its Receptor Signaling Complex to Initiate CD4 T Lymphocyte Response. J. Biol. Chem..

[14] McElroy C.A., Holland P.J., Zhao P., Lim J.-M., Wells L., Eisenstein E., Walsh S.T.R. (2012). Structural reorganization of the interleukin-7 signaling complex. Proc. Natl. Acad. Sci. USA.

[15] Jiang Q., Li W.Q., Hofmeister R.R., Young H.A., Hodge D.R., Keller J.R., Khaled A.R., Durum S.K. (2004). Distinct Regions of the Interleukin-7 Receptor Regulate Different Bcl2 Family Members. Mol. Cell. Biol..

[16] Jiang Q., Li W.Q., Aiello F.B., Mazzucchelli R., Asefa B., Khaled A.R., Durum S.K. (2005). Cell biology of IL-7, a key lymphotrophin. Cytokine Growth Factor Rev..

[17] Jiang Q., Li W.-Q., Aiello F.B., Klarmann K.D., Keller J.R., Durum S.K. (2005). Retroviral transduction of IL-7Rα into IL-7Rα−/− bone marrow progenitors: Correction of lymphoid deficiency and induction of neutrophilia. Gene Ther..

[18] Palmer M.J., Mahajan V.S., Trajman L.C., Irvine D.J., Lauffenburger D.A., Chen J. (2008). Interleukin-7 Receptor Signaling Network: An Integrated Systems Perspective. Cell. Mol. Immunol..

[19] Venkitaraman A.R., Cowling R.J. (1994). Interleukin-7 induces the association of phosphatidylinositol 3-kinase with the α chain of the interleukin-7 receptor. Eur. J. Immunol..

[20] Crawley J.B., Willcocks J., Foxwell B.M.J. (1996). Interleukin-7 induces T cell proliferation in the absence of Erk/MAP kinase activity. Eur. J. Immunol..

[21] Osborne L.C., Dhanji S., Snow J.W., Priatel J.J., Ma M.C., Miners M.J., Teh H.-S., Goldsmith M.A., Abraham N. (2007). Impaired CD8 T cell memory and CD4 T cell primary responses in IL-7Rα mutant mice. J. Exp. Med..

[22] Canté-Barrett K., Spijkers-Hagelstein J.A.P., Buijs-Gladdines J.G.C.A.M., Uitdehaag J.C.M., Smits W.K., van der Zwet J., Buijsman R.C., Zaman G.J.R., Pieters R., Meijerink J.P.P. (2016). MEK and PI3K-AKT inhibitors synergistically block activated IL7 receptor signaling in T-cell acute lymphoblastic leukemia. Leukemia.

[23] Bousoik E., Montazeri Aliabadi H. (2018). “Do We Know Jack” About JAK? A Closer Look at JAK/STAT Signaling Pathway. Front. Oncol..

[24] Winston L.A., Hunter T. (1996). Intracellular signalling: Putting JAKs on the kinase MAP. Curr. Biol..

[25] Pandey A., Ozaki K., Baumann H., Levin S.D., Puel A., Farr A.G., Ziegler S.F., Leonard W.J., Lodish H.F. (2000). Cloning of a receptor subunit required for signaling by thymic stromal lymphopoietin. Nat. Immunol..

[26] Levin S.D., Koelling R.M., Friend S.L., Isaksen D.E., Ziegler S.F., Perlmutter R.M., Farr A.G. (1999). Thymic stromal lymphopoietin: A cytokine that promotes the development of IgM+ B cells in vitro and signals via a novel mechanism. J. Immunol..

[27] Isaksen D.E., Baumann H., Trobridge P.A., Farr A.G., Levin S.D., Ziegler S.F. (1999). Requirement for stat5 in thymic stromal lymphopoietin-mediated signal transduction. J. Immunol..

[28] McElroy C.A., Dohm J.A., Walsh S.T.R. (2009). Structural and Biophysical Studies of the Human IL-7/IL-7Rα Complex. Structure.

[29] Pillet A.-H., Juffroy O., Mazard-Pasquier V., Moreau J.-L., Gesbert F., Chastagner P., Colle J.-H., Thèze J., Rose T. (2008). Human IL-Rbeta chains form IL-2 binding homodimers. Eur. Cytokine Netw..

[30] Tamarit B., Bugault F., Pillet A.-H., Lavergne V., Bochet P., Garin N., Schwarz U., Thèze J., Rose T. (2013). Membrane Microdomains and Cytoskeleton Organization Shape and Regulate the IL-7 Receptor Signalosome in Human CD4 T-cells. J. Biol. Chem..

[31] Henriques C.M., Rino J., Nibbs R.J., Graham G.J., Barata J.T. (2010). IL-7 induces rapid clathrin-mediated internalization and JAK3-dependent degradation of IL-7Rα in T cells. Blood.

[32] Dooms H. (2013). Interleukin-7: Fuel for the autoimmune attack. J. Autoimmun..

[33] Giliani S., Mori L., de Saint Basile G., Le Deist F., Rodriguez-Perez C., Forino C., Mazzolari E., Dupuis S., Elhasid R., Kessel A. (2005). Interleukin-7 receptor alpha (IL-7Ralpha) deficiency: Cellular and molecular bases. Analysis of clinical, immunological, and molecular features in 16 novel patients. Immunol. Rev..

[34] Ye S.K., Maki K., Kitamura T., Sunaga S., Akashi K., Domen J., Weissman I.L., Honjo T., Ikuta K. (1999). Induction of germline transcription in the TCRγ, locus by Stat5: Implications for accessibility control by the IL-7 receptor. Immunity.

[35] Puel A., Ziegler S.F., Buckley R.H., Leonard W.J. (1998). Defective IL7R expression in T-B+NK+ severe combined immunodeficiency. Nat. Genet..

[36] Roifman C.M., Zhang J., Chitayat D., Sharfe N. (2000). A partial deficiency of interleukin-7R alpha is sufficient to abrogate T-cell development and cause severe combined immunodeficiency. Blood.

[37] Buckley R.H. (2002). Primary cellular immunodeficiencies. J. Allergy Clin. Immunol..

[38] Kwan A., Abraham R.S., Currier R., Brower A., Andruszewski K., Abbott J.K., Baker M., Ballow M., Bartoshesky L.E., Bonilla F.A. (2014). Newborn screening for severe combined immunodeficiency in 11 screening programs in the United States. JAMA J. Am. Med. Assoc..

[39] Giliani S., Bonfim C., de Saint Basile G., Lanzi G., Brousse N., Koliski A., Malvezzi M., Fischer A., Notarangelo L.D., Le Deist F. (2006). Omenn syndrome in an infant with IL7RA gene mutation. J. Pediatr..

[40] Lebet T., Chiles R., Hsu A.P., Mansfield E.S., Warrington J.A., Puck J.M. (2008). Mutations causing severe combined immunodeficiency: Detection with a custom resequencing microarray. Genet. Med..

[41] Lev A., Simon A.J., Barel O., Eyal E., Glick-Saar E., Nayshool O., Birk O., Stauber T., Hochberg A., Broides A. (2019). Reduced Function and Diversity of T Cell Repertoire and Distinct Clinical Course in Patients With IL7RA Mutation. Front. Immunol..

[42] Engelhardt K.R., Xu Y., Grainger A., Germani Batacchi M.G.C., Swan D.J., Willet J.D.P., Abd Hamid I.J., Agyeman P., Barge D., Bibi S. (2017). Identification of Heterozygous Single- and Multi-exon Deletions in IL7R by Whole Exome Sequencing. J. Clin. Immunol..

[43] Lundtoft C., Awuah A.A.-A., Güler A., Harling K., Schaal H., Mayatepek E., Phillips R.O., Nausch N., Owusu-Dabo E., Jacobsen M. (2019). An IL7RA exon 5 polymorphism is associated with impaired IL-7Rα splicing and protection against tuberculosis in Ghana. Genes Immun..

[44] Hahn W.-H., Suh J.-S., Park H.-J., Cho B.-S. (2011). Interleukin 7 receptor gene polymorphisms and haplotypes are associated with susceptibility to IgA nephropathy in Korean children. Exp. Ther. Med..

[45] Gallego-Bustos F., Gotea V., Ramos-Amador J.T., Rodríguez-Pena R., Gil-Herrera J., Sastre A., Delmiro A., Rai G., Elnitski L., González-Granado L.I. (2016). A Case of IL-7R Deficiency Caused by a Novel Synonymous Mutation and Implications for Mutation Screening in SCID Diagnosis. Front. Immunol..

[46] Bayer D.K., Martinez C.A., Sorte H.S., Forbes L.R., Demmler-Harrison G.J., Hanson I.C., Pearson N.M., Noroski L.M., Zaki S.R., Bellini W.J. (2014). Vaccine-associated varicella and rubella infections in severe combined immunodeficiency with isolated CD4 lymphocytopenia and mutations in IL 7 R detected by tandem whole exome sequencing and chromosomal microarray. Clin. Exp. Immunol..

[47] Shamim Z., Spellman S., Haagenson M., Wang T., Lee S.J., Ryder L.P., Müller K. (2013). Polymorphism in the Interleukin-7 Receptor-alpha and Outcome after Allogeneic Hematopoietic Cell Transplantation with Matched Unrelated Donor. Scand. J. Immunol..

[48] Liao C.-Y., Yu H.-W., Cheng C.-N., Chen J.-S., Lin C.-W., Chen P.-C., Shieh C.-C. (2018). A novel pathogenic mutation on Interleukin-7 receptor leading to severe combined immunodeficiency identified with newborn screening and whole exome sequencing. J. Microbiol. Immunol. Infect..

[49] Lundmark F., Duvefelt K., Iacobaeus E., Kockum I., Wallström E., Khademi M., Oturai A., Ryder L.P., Saarela J., Harbo H.F. (2007). Variation in interleukin 7 receptor α chain (IL7R) influences risk of multiple sclerosis. Nat. Genet..

[50] Gregory S.G., Schmidt S., Seth P., Oksenberg J.R., Hart J., Prokop A., Caillier S.J., Ban M., Goris A., Barcellos L.F. (2007). Interleukin 7 receptor α chain ( IL7R ) shows allelic and functional association with multiple sclerosis. Nat. Genet..

[51] Todd J.A., Walker N.M., Cooper J.D., Smyth D.J., Downes K., Plagnol V., Bailey R., Nejentsev S., Field S.F., Payne F. (2007). Robust associations of four new chromosome regions from genome-wide analyses of type 1 diabetes. Nat. Genet..

[52] O’Doherty C., Alloza I., Rooney M., Vandenbroeck K. (2009). IL7RA polymorphisms and chronic inflammatory arthropathies. Tissue Antigens.

[53] Zhang Z., Duvefelt K., Svensson F., Masterman T., Jonasdottir G., Salter H., Emahazion T., Hellgren D., Falk G., Olsson T. (2005). Two genes encoding immune-regulatory molecules (LAG3 and IL7R) confer susceptibility to multiple sclerosis. Genes Immun..

[54] Heron M., Grutters J.C., van Moorsel C.H.M., Ruven H.J.T., Huizinga T.W.J., van der Helm-van Mil A.H.M., Claessen A.M.E., van den Bosch J.M.M. (2009). Variation in IL7R predisposes to sarcoid inflammation. Genes Immun..

[55] Bodian D.L., McCutcheon J.N., Kothiyal P., Huddleston K.C., Iyer R.K., Vockley J.G., Niederhuber J.E. (2014). Germline Variation in Cancer-Susceptibility Genes in a Healthy, Ancestrally Diverse Cohort: Implications for Individual Genome Sequencing. PLoS ONE.

[56] Genain C.P., Cannella B., Hauser S.L., Raine C.S. (1999). Identification of autoantibodies associated with myelin damage in multiple sclerosis. Nat. Med..

[57] Hafler D.A., Compston A., Sawcer S., Lander E.S., Daly M.J., De Jager P.L., De Bakker P.I.W., Gabriel S.B., Mirel D.B., Ivinson A.J. (2007). Risk Alleles for Multiple Sclerosis Identified by a Genomewide Study. N. Engl. J. Med..

[58] Cox A.L., Thompson S.A.J., Jones J.L., Robertson V.H., Hale G., Waldmann H., Compston D.A.S., Coles A.J. (2005). Lymphocyte homeostasis following therapeutic lymphocyte depletion in multiple sclerosis. Eur. J. Immunol..

[59] Traggiai E., Biagioli T., Rosati E., Ballerini C., Mazzanti B., Ben Nun A., Massacesi L., Vergelli M. (2001). IL-7-enhanced T-cell response to myelin proteins in multiple sclerosis. J. Neuroimmunol..

[60] Galarza-Muñoz G., Briggs F.B.S., Evsyukova I., Schott-Lerner G., Kennedy E.M., Nyanhete T., Wang L., Bergamaschi L., Widen S.G., Tomaras G.D. (2017). Human Epistatic Interaction Controls IL7R Splicing and Increases Multiple Sclerosis Risk. Cell.

[61] Zenatti P.P., Ribeiro D., Li W., Zuurbier L., Silva M.C., Paganin M., Tritapoe J., Hixon J.A., Silveira A.B., Cardoso B.A. (2011). Oncogenic IL7R gain-of-function mutations in childhood T-cell acute lymphoblastic leukemia. Nat. Genet..

[62] Shochat C., Tal N., Bandapalli O.R., Palmi C., Ganmore I., te Kronnie G., Cario G., Cazzaniga G., Kulozik A.E., Stanulla M. (2011). Gain-of-function mutations in interleukin-7 receptor -α ( IL7R ) in childhood acute lymphoblastic leukemias. J. Exp. Med..

[63] Shochat C., Tal N., Gryshkova V., Birger Y., Bandapalli O.R., Cazzaniga G., Gershman N., Kulozik A.E., Biondi A., Mansour M.R. (2014). Novel activating mutations lacking cysteine in type I cytokine receptors in acute lymphoblastic leukemia. Blood.

[64] Huh H.J., Lee S.H., Yoo K.H., Sung K.W., Koo H.H., Jang J.H., Kim K., Kim S.J., Kim W.S., Jung C.W. (2013). Gene mutation profiles and prognostic implications in Korean patients with T-lymphoblastic leukemia. Ann. Hematol..

[65] Kim M.S., Chung N.G., Kim M.S., Yoo N.J., Lee S.H. (2013). Somatic mutation of IL7R exon 6 in acute leukemias and solid cancers. Hum. Pathol..

[66] Richter-Pechańska P., Kunz J.B., Hof J., Zimmermann M., Rausch T., Bandapalli O.R., Orlova E., Scapinello G., Sagi J.C., Stanulla M. (2017). Identification of a genetically defined ultra-high-risk group in relapsed pediatric T-lymphoblastic leukemia. Blood Cancer J..

[67] Zhang J., Ding L., Holmfeldt L., Wu G., Heatley S.L., Payne-Turner D., Easton J., Chen X., Wang J., Rusch M. (2012). The genetic basis of early T-cell precursor acute lymphoblastic leukaemia. Nature.

[68] Roberts K.G., Li Y., Payne-Turner D., Harvey R.C., Yang Y.-L., Pei D., McCastlain K., Ding L., Lu C., Song G. (2014). Targetable Kinase-Activating Lesions in Ph-like Acute Lymphoblastic Leukemia. N. Engl. J. Med..

[69] Roberts K.G., Morin R.D., Zhang J., Hirst M., Zhao Y., Su X., Chen S.-C., Payne-Turner D., Churchman M.L., Harvey R.C. (2012). Genetic Alterations Activating Kinase and Cytokine Receptor Signaling in High-Risk Acute Lymphoblastic Leukemia. Cancer Cell.

[70] Roberts K.G., Yang Y.-L., Payne-Turner D., Lin W., Files J.K., Dickerson K., Gu Z., Taunton J., Janke L.J., Chen T. (2017). Oncogenic role and therapeutic targeting of ABL-class and JAK-STAT activating kinase alterations in Ph-like ALL. Blood Adv..

[71] Rozovski U., Li P., Harris D., Ohanian M., Kantarjian H., Estrov Z. (2014). Interleukin-7 receptor- α gene mutations are not detected in adult T-cell acute lymphoblastic leukemia. Cancer Med..

[72] Hixon J.A., Andrews C., Kashi L., Kohnhorst C.L., Senkevitch E., Czarra K., Barata J.T., Li W., Schneider J.P., Walsh S.T.R. (2019). New anti-IL-7Rα monoclonal antibodies show efficacy against T cell acute lymphoblastic leukemia in pre-clinical models. Leukemia.

[73] Akkapeddi P., Fragoso R., Hixon J.A., Ramalho A.S., Oliveira M.L., Carvalho T., Gloger A., Matasci M., Corzana F., Durum S.K. (2019). A fully human anti-IL-7Rα antibody promotes antitumor activity against T-cell acute lymphoblastic leukemia. Leukemia.

[74] Mendes R.D., Sarmento L.M., Cante-Barrett K., Zuurbier L., Buijs-Gladdines J.G.C.A.M., Povoa V., Smits W.K., Abecasis M., Yunes J.A., Sonneveld E. (2014). PTEN microdeletions in T-cell acute lymphoblastic leukemia are caused by illegitimate RAG-mediated recombination events. Blood.

[75] Papaemmanuil E., Rapado I., Li Y., Potter N.E., Wedge D.C., Tubio J., Alexandrov L.B., Van Loo P., Cooke S.L., Marshall J. (2014). RAG-mediated recombination is the predominant driver of oncogenic rearrangement in ETV6-RUNX1 acute lymphoblastic leukemia. Nat. Genet..

[76] Kirkham C.M., Scott J.N.F., Wang X., Smith A.L., Kupinski A.P., Ford A.M., Westhead D.R., Stockley P.G., Tuma R., Boyes J. (2019). Cut-and-Run: A Distinct Mechanism by which V(D)J Recombination Causes Genome Instability. Mol. Cell.

[77] Stroud R.M., Wells J.A. (2004). Mechanistic Diversity of Cytokine Receptor Signaling Across Cell Membranes. Sci. Signal..

[78] Lu X., Gross A.W., Lodish H.F. (2006). Active Conformation of the Erythropoietin Receptor. J. Biol. Chem..

[79] Brooks A.J., Dai W., O’Mara M.L., Abankwa D., Chhabra Y., Pelekanos R.A., Gardon O., Tunny K.A., Blucher K.M., Morton C.J. (2014). Mechanism of Activation of Protein Kinase JAK2 by the Growth Hormone Receptor. Science.

[80] Liu Y., Easton J., Shao Y., Maciaszek J., Wang Z., Wilkinson M.R., McCastlain K., Edmonson M., Pounds S.B., Shi L. (2017). The genomic landscape of pediatric and young adult T-lineage acute lymphoblastic leukemia. Nat. Genet..

[81] Porcu M., Kleppe M., Gianfelici V., Geerdens E., De Keersmaecker K., Tartaglia M., Foà R., Soulier J., Cauwelier B., Uyttebroeck A. (2012). Mutation of the receptor tyrosine phosphatase PTPRC (CD45) in T-cell acute lymphoblastic leukemia. Blood.

[82] Weijenborg Campos L., Pini Zenatti P., Granato Pissinato L., Libanio Rodrigues G.O., Artico L.L., Rafael Guimarães T., Fröhlich Archangelo L., Martínez L., Brooks A.J., Yunes J.A. (2019). Oncogenic basic amino acid insertions at the extracellular juxtamembrane region of IL7RA cause receptor hypersensitivity. Blood.

[83] Fry T.J., Mackall C.L. (2005). The Many Faces of IL-7: From Lymphopoiesis to Peripheral T Cell Maintenance. J. Immunol..

[84] Silva A., Laranjeira A.B.A., Martins L.R., Cardoso B.A., Demengeot J., Yunes J.A., Seddon B., Barata J.T. (2011). IL-7 Contributes to the Progression of Human T-cell Acute Lymphoblastic Leukemias. Cancer Res..

